# Identification and Quantification of Anthocyanin and Catechin Compounds in Purple Tea Leaves and Flakes

**DOI:** 10.3390/molecules27196676

**Published:** 2022-10-07

**Authors:** El-Sayed M. Abdel-Aal, Iwona Rabalski, Lili Mats, Ishan Rai

**Affiliations:** 1 Agriculture and Agri-Food Canada, Guelph Research and Development Centre, 93 Stone Road West, Guelph, ON N1G 5C9, Canada; 2 Asilia Inc., Mississauga, ON L5L 5Y7, Canada & Houston, TX 77073, USA

**Keywords:** purple and green tea, nutrients, polyphenols, UPLC, LC-MS/MS

## Abstract

Tea is the first most popular beverage worldwide and is available in several selections such as black (fully oxidized), Oolong (partially oxidized) and green (non-oxidized), in addition to purple tea, an emerging variety derived from the same tea plant (*Camellia sinensis*). This study investigated purple tea leaves (non-oxidized) and flakes (water extractable) to thoroughly identify their composition of anthocyanins and catechins and to study the effect of a water extraction process on their compositional properties in comparison with green tea. Anthocyanin and catechin compounds were separated and quantified using UPLC, and their identity was confirmed using LC-MS/MS in positive and negative ionization modes. Delphinidin was the principal anthocyaninidin in purple tea, while cyanidin came in second. The major anthocyanin pigments in purple tea were delphinidin-coumaroyl-hexoside followed by delphinidin-3-galactoside and cyanidin-coumaroyl-hexoside. The water extraction process resulted in substantial reductions in anthocyanins in purple tea flakes. There were no anthocyanin compounds detected in green tea samples. Both purple and green tea types were rich in catechins, with green tea containing higher concentrations than purple tea. The main catechin in purple or green tea was epigallocatechin gallate (EGCG) followed by either epicatechin gallate (ECG) or epigallocatechin (EGC), subject to tea type. The extraction process increased the concentration of catechins in both purple and green tea flakes. The results suggest that purple tea holds promise in making healthy brews, natural colorants and antioxidants and/or functional ingredients for beverages, cosmetics and healthcare industries due to its high content of anthocyanins and catechins.

## 1. Introduction

Tea (*Camellia sinensis*) is an important cash crop whose leaves are processed into the most consumed drink in the world after water [1]. Tea is available in several beverage choices such as black (fully oxidized), Oolong (partially oxidized) and green (non-oxidized). Additionally, an emerging purple tea variety derived from the same tea plant is now commercially produced as a healthy beverage. In general, tea—especially green tea—is considered a healthy beverage due to its high content of polyphenols which have demonstrated potential positive health effects via their roles as antioxidants [2,3,4,5,6], immune stimulators and anti-cancer [7], anti-obesity and anti-diabetic [8,9,10], anti-alcoholism [11], anti-hypertension [12] and anticardiovascular [13] diseases. The composition of polyphenols varies among teas and is influenced by a number of factors including tea variety, growing environment and processing method [14].

Catechins, a group of secondary plant metabolites belonging to the flavan-3-ols (a subclass of flavonoids), are the major polyphenols in green tea and are responsible for its health claims [15]. They are considered safe for human consumption, with epigallocatechin-3-gallate (EGCG) being the most abundant catechin in green tea brew with a daily intake of 90–300 mg [16]. Catechins scavenge reactive oxygen species and chelate metal ions, in addition to their indirect antioxidant activities via the induction of antioxidant enzymes, inhibition of pro-oxidant enzymes and production of phase II detoxification enzymes [17]. Research has shown that tea polyphenols have been employed in the prevention and treatment of diseases such as neurodegenerative, cardiovascular and cerebrovascular, cancer, diabetes, high blood pressure and scurvy diseases [18]. They exert their antioxidant activities primarily by regulating the Nrf2 signaling pathway and stimulating the NF-kB and MAPK pathways to balance cellular redox status.

In addition to catechins, the emerging purple tea variety contains another polyphenol category, anthocyanins [2,5,11]. Anthocyanins are water-soluble pigments that impart colorful red, purple or blue hue to fruits, vegetables, leaves, flowers and grains. They play crucial roles in plants via enhancing their survivability as free radical scavengers and light filters besides their contribution to pollination and seed dispersal as attractants for insects [19,20,21]. They are also important components of the human diet with a daily intake of 12.5 mg in the United States [22] and 19.8–64.9 mg in men and 18.4–44.1 mg in women in Europe [23]. Their daily consumption varies among countries and regions subject to type of diet and gender. Currently, there is a body of evidence that demonstrates their roles in human health as antioxidant, anti-inflammatory, anti-diabetic and anti-cancer ingredients [24,25]. The composition of anthocyanin in purple tea varies among varieties subject to genotype and growing environment with delphinidin-based or cyanidin-related anthocyanins being the most abundant [5,26,27]. Since little data on anthocyanin composition in purple tea are available, the current study was intended to explore the thorough composition of anthocyanin pigments in purple tea leaves and flakes. In addition, the composition of catechins and other bioactive compounds in purple tea leaves and flakes was also investigated and compared with their counterparts in green tea. Moreover, the effect of processing purple tea into flakes on its composition of anthocyanins and catechins was assessed in comparison with green tea flakes.

## 2. Materials and Methods

### 2.1. Chemicals

Nine HPLC grade authentic anthocyanin standards including cyanidin-3,5-diglucoside (Cy-3,5-diGlu), cyanidin-3-galactoside (Cy-3-Gal), cyanidin-3-glucoside (Cy-3-Glu), cyanidin-3-rutinoside (Cy-3-Rut), delphinidin-3-glucoside (Dp-3-Glu), malvidin-3-glucoside, (Mv-3-Glu), pelargonidin-3-glucoside (Pg-3-Glu), peonidin-3-glucoside (Pn-3-Glu) and petunidin-3-glucoside (Pt-3-Glu) were purchased from Polyphenols Laboratories AS (Sandnes, Norway). Seven HPLC grade authentic catechin standards as per the ISO method 14502-2 including (+)-catechin (C, 43412), (−)-epicatechin (EC, 03950490), (−)-epigallocatechin (EGC, E3768), (−)-epigallocatechin gallate (EGCG, E4143), (−)-epicatechin gallate (ECG, E3893), (−)-gallocatechin (GC, G6657) and (−)-gallocatechingallate (GCG, G6782) were purchased from Sigma Aldridge (Oakville, ON, Canada) along with caffeine (C1778-1VL), theobromine (T4500) and gallic acid (91215). Other HPLC chemicals and solvents were purchased either from Fisher Scientific Canada or VWR Canada. Nano pure water was obtained from Milli-Q integral water purification system (Millipore (Canada) Ltd., Etobicoke, ON, Canada).

### 2.2. Tea Samples

The investigated tea samples were provided by Asilia Inc. (Mississauga, ON, Canada). The samples were random and representative lots of purple tea leaves and flakes and two lots of green tea leaves and flakes. The green tea samples were included in the study as a reference material. Both purple and green tea leaves were unfermented and non-oxidized dried products, while tea flakes were water extractable minimally processed products. The tea leaves and flakes were milled into a powder form using a laboratory mill to obtain uniform and homogenous ground materials for analysis.

### 2.3. Analysis of Nutrients

Moisture and ash in tea samples were determined according to the AOAC methods 925.10 and 923.03, respectively [28]. Moisture is based on drying the samples in an air oven at 130 °C for 1 h, while ash is determined by dry ashing the samples in a furnace at 525 °C for 8 h. The protein was determined based on the high temperature combustion method using a Nitrogen Analyzer (Flash 2000, Thermo Fisher Scientific, Waltham, MA, USA). The content of nitrogen was converted into protein using the conversion factor 6.25.

### 2.4. Analysis of Anthocyanins

**Figure 1:**
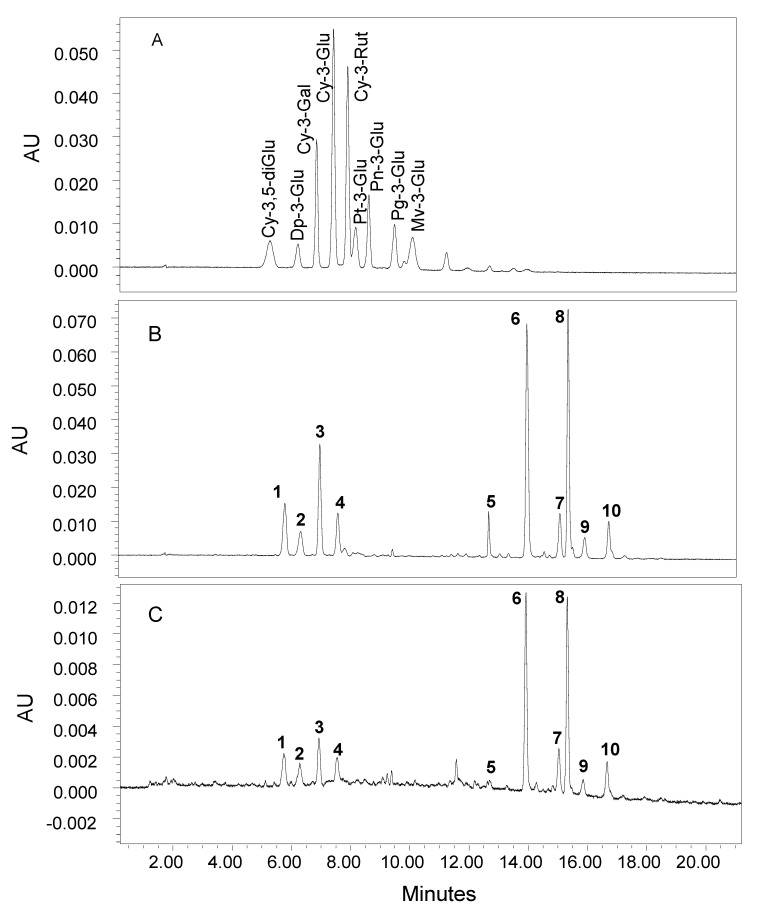
Typical UPLC chromatograms of a standard mix of 9 authentic anthocyanin compounds (**A**) and samples of purple tea leaves (**B**) and flakes (**C**). See Table 1 for identity of peak numbers.

Anthocynins were extracted from purple and green tea samples with methanol acidified with formic acid at 80:20 (*v*/*v*). Then, 5 mL of acidified solvent was added to accurately weighed tea sample of 60–90 mg. The samples were extracted for 30 min using an orbital IKA Vibrax VXR stirrer (Janke & Kunkel Co., Staunfen, Germany) at 1800 rpm. The extracts were centrifuged for 10 min at 4400 rpm (Sorvall ST-8 centrifuge, Thermo Fisher Scientific, Nepean, ON, Canada). The extraction was repeated, and the acidified extracts were pooled. The ground tea flakes were dissolved directly in 5 mL of 2% formic acid and then vortexed and sonicated for 30 s. All samples were filtered through a 0.2 μm GHP syringe filter (Paill Gelman Laboratory, Ann Arbor, MI, USA) prior to analysis. The separation, quantification and identification of anthocyanin compounds were performed on UPLC system equipped with PDA and MS detectors (Acquity H class, Waters, Mississauga, ON, Canada). The anthocyanin compounds were separated on Agilent Zorbax SB-C18 column (2.1 × 150 mm, 1.8 µm micron particle size) using quaternary solvent system, which was composed of 2% formic acid (solvent A) and 100% acetonitrile (solvent B) at 0.25 mL/min flow rate. The gradient was programmed as follows: 0 min 7% B, 12 min 20% B, 28 min 30% B, 29 min 0% B, 32 min 0% B, 33 min 93%B and 39 min 93% B, and the remaining percent is A. The separated anthocyanin compounds were identified and quantified based on their absorbance at 525 nm and mass using PDA and MS-QDa detectors and compared with anthocyanin standards if available. The mass spectrometry detector was set in the ESI positive polarity, gain 1, capillary 1.5 V, cone 15 V, probe 350 °C and sampling rate 8 points/second. The MS-QDa was set in advance mode in which SIR(s) was set based on molecular ions of the investigated compounds. A Typical UPLC chromatogram depicting the separation of a standard mix of 9 authentic anthocyanins is presented in Figure 1A.

For further identification and confirmation of anthocyanin compounds, LC-MS/MS analysis was performed on a Thermo^®^ Scientific Q-Exactive™ Orbitrap mass spectrometer connected to a Vanquish™ Flex Binary UPLC System (Waltham, MA, USA). The same Agilent Zorbax SB-C18 column and UPLC conditions used in Waters system was applied in the LC-MS/MS analysis. Positive heated electrospray ionization (ESI) mode was used for the majority of this study, and the negative ESI mode was used to obtain alternative fragmentation to confirm the presence of coumaroyl-hexosides. Mass spectrometry data were collected using either Full-MS/DDMS2 (TopN = 10, NCE = 15) or PRM (NCE = 15 for positive, NCE = 15, 25 for negative) modes. Data were visualized and analysed using Thermo FreeStyle™ 1.7 software.

### 2.5. Analysis of Catechins

**Figure 2:**
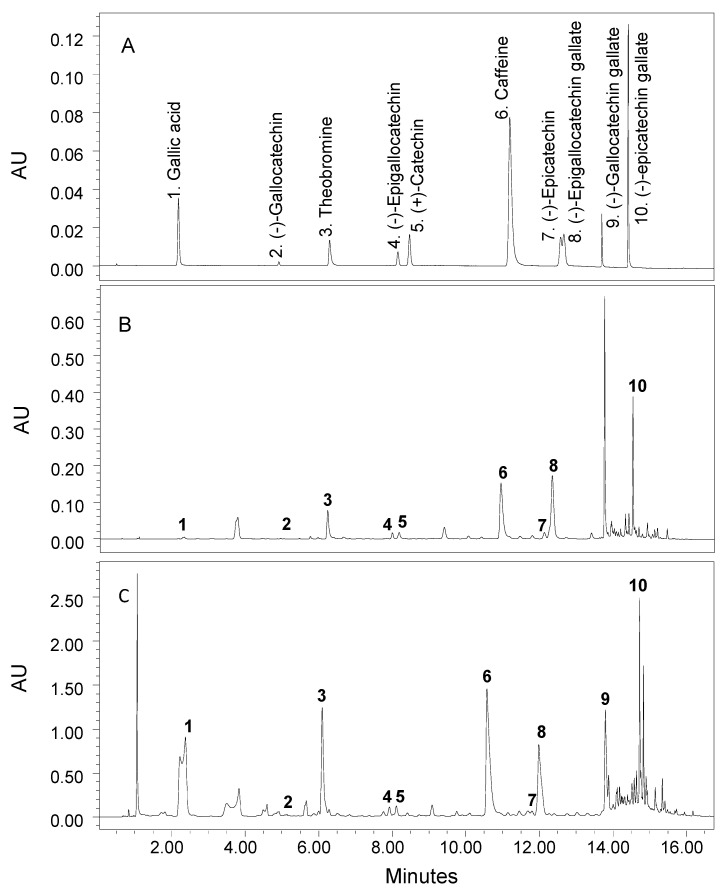
Typical UPLC chromatograms of a standard mix of authentic 7 catechin and other 3 bioactive compounds (**A**) and samples of purple tea leaves (**B**) and flakes (**C**).

Extraction, separation and quantification of catechins were based on the ISO method (ISO 14502-2) with some changes to suit the mass spectrometry system and in order to utilize the same acidified methanol extracts for the determination of catechins and anthocyanins as well. These changes include the withdrawal of chemical modifiers and replacement of acetic acid with formic acid, as it is more suitable for mass spectrometry. The heating of acidified extracts was also excluded. The tea samples were extracted as per the above anthocyanin extraction method. The analysis of catechins and other three bioactive compounds was performed on UPLC equipped with PDA and MS detectors. The mobile phase was composed of 2% formic acid (A) and acetonitrile (B), and the compounds were separated on Zorbax SB-C18 column at 35 °C and 0.45 mL/min flow rate with the following gradient program: 0 min 5% B, 8 min 10% B, 12 min 12% B, 16 min 30% B, 17 min 100% B, 20 min 100% B, 21 min 5% B and 27 min 5% B, and the remaining percent is A. The separated catechin compounds were detected with PDA detector at 275 nm, and their identity was confirmed with MS-QDa detector in ESI positive mode using the same settings as in the analysis of anthocyanins with the use of appropriate molecular ions for catechins. The separated catechins were quantified based on their corresponding catechin standard calibration curves. A typical UPLC chromatogram showing the separation of a standard mix of authentic catechin compounds is shown in Figure 2A.

### 2.6. Statistical Analysis

Data were subjected to one-way ANOVA to determine differences between tea lots. Significant differences between means were assessed using Tukey method and considered significant at *p* < 0.05. The data are expressed as means ± SD (standard deviation). Statistical analyses were performed using Sigma-Plot version 14.5 (Systat Software Inc., San Jose, CA, USA).

## 3. Results and Discussion

### 3.1. Nutrient Content

Purple and green tea leaves and flakes exhibited low moisture content ranging from 5.9 to 6.3% (Table 1). On average, the leaves had slightly higher moisture levels (6.1 and 6.3%) compared with flakes (5.9 and 6.0%) for green and purple tea, respectively. These results indicate that both tea products have a suitable moisture level for safe storage and prolonged shelf life. The purple and green tea products had reasonable amounts of total ash content (Table 1). In a previous study, green and black tea leaves contained about 6.1–6.2% total ash [29] which was close to the current results. The water extraction process resulted in concentrating minerals in the resultant flakes due to the solubility of minerals in water. The tea flakes could be considered a good source of minerals at levels of 8.6–9.2%. In general, tea is considered a useful source of some minerals in the human diet, especially potassium, magnesium and manganese [30]. The tea leaves and flakes also contained rational amounts of protein ranging from 17.5–25.4% on average (Table 1). Contrarily to the minerals, the protein content in flakes was lower than that in their corresponding tea leaves. This indicates that a part of the tea proteins was lost during the extraction process, possibly the non-extractable fraction. Research has shown that tea leaves contain approximately 21–28% [31] or 24% protein [29], with glutamic acid being the most abundant amino acid in tea protein followed by aspartic acid, leucine, lysine, alanine and valine [32]. Only few significant differences were found among the lots of tea flakes in terms of ash and protein content, possibly due to processing conditions. The current results suggest that purple tea flakes can be considered a good source of minerals and protein for use in food and non-food applications.

### 3.2. Composition of Anthocyanins

More than 20 anthocyanin pigments were detected in purple tea, of which 10 anthocyanin compounds were present at fairly high concentrations; therefore, they are considered major ones for further identification and quantification (Figure 1B,C). The 10 major anthocyanin compounds were separated into two clusters, the first one has four anthocyanin pigments (peaks 1–4), while the second group comprises of six anthocyanins (peaks 5–10). The first cluster contains the non-acylated anthocyanins while the second group has the acylated ones. Other studies have shown the presence of the non-acylated and acylated anthocyanins, but they exhibited distinct compositions due to the differences in genotype and growing environment [2,5,33,34,35]. In other words, the anthocyanin profile in purple tea is quite similar, but purple teas are different in the content and composition of individual anthocyanin pigments. In this study, anthocyanin compounds were characterized on the basis of UV–Vis and MS properties and the congruence of their retention times compared with authentic anthocyanins. Wherever possible, confirmation of identity was achieved by the congruence of these properties with those of authentic anthocyanin standards. Additionally, the identity of anthocyanin compounds was also confirmed by MS/MS analysis. The MS fragmentation of anthocyanin often shows two major ions: the molecular ion [M]^+^ and a fragment ion [M-X]^+^ arising from the loss of the sugar moiety (Supplementary Figure S1). The MS and UV–Vis characteristics of the 10 major anthocyanins are presented in Table 2. The primary aglycone or anthocyanidin found in purple tea was delphinidin (*m/z* 303) with delphinidin-based anthocyanins constituting about 78% of the total major anthocyanins (Table 2). Cyanidin, *m/z* 287 and petunidin, *m/z* 317 were also detected in the major anthocyanins.

**Figure 3:**
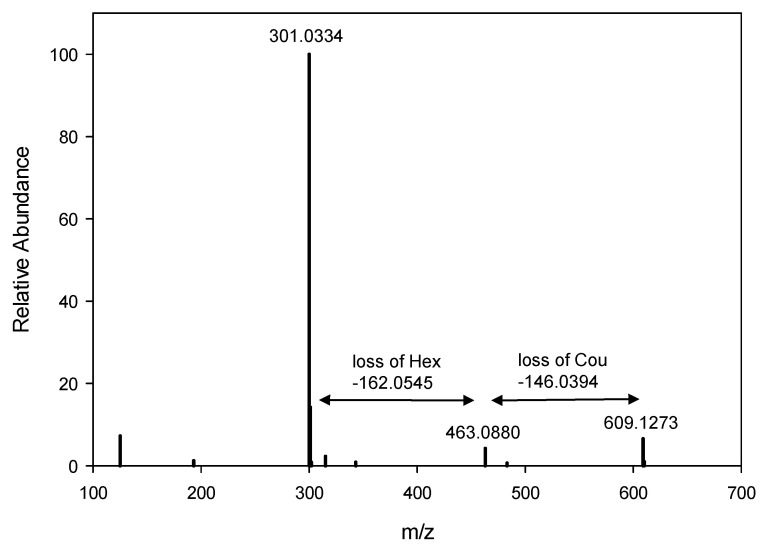
MS fragmentation spectrum of Dp-Cou-Hex in the ESI negative ionization mode shows an additional fragment at *m*/*z* = 463.0880, which confirms the presence of the coumaroyl moiety.

The identity of anthocyanin pigments in the first cluster was confirmed on the basis of the congruence of properties with authentic standards; thus, they were totally identified (Table 2). They are delphinidin-3-galactoside (Dp-3-Gal), delphinidin-3-glucoside (Dp-3-Glu), cyanidin-3-galactoside (Cy-3-Gal) and cyanidin-3-glucoside (Cy-3-Glu). The acylated anthocyanins in the second group (peaks 5–10) were identified based on the analogy of the chromatographic, UV and MS properties. They are delphinidin-coumaroyl-hexoside (Dp-Cou-Hex) isomer 1, 2 and 3, cyanidin-coumaroyl-hexoside (Cy-Cou-Hex) isomer 1 and 2 and petunidin-coumaroyl-hexoside (Pt-Cou-Hex). The identity of the courmoyl moiety was confirmed based on MS/MS ESI in the negative mode showing the presence of an additional fragment at m/z = 463, which confirms the loss of the coumaroyl fragment (Figure 3). The hexose could be either galactose or glucose, which was detected and confirmed in the non-acylated anthocyanins. Further research is needed to confirm the type of sugar for each pigment and its exact bond position. A study on the composition of anthocyanin in the purple tea variety, Zijuan, the most common anthocyanin-rich tea cultivar in China, has shown the presence of eight anthocyanins, pelargonidin-3,5-diglucoside, cyanidin-3-galactoside, cyanidin-3-glucoside, delphinidin, cyanidin, pelargonidin, peonidin, and malvidin, with cyanidin-3-galactoside being the most abundant [5]. In another study, only delphinidin, cyanidin and pelargonidin (882 µg/g) were found in Chinese purple tea cv. Ziyan, with delphinidin (708 µg/g) being the predominant [34]. The composition of anthocyanidins in Kenyan purple tea from the highest to lowest has been reported to be cyanidin, pelargonidin, peonidin, malvidin and finally delphinidin [35], whereas other Kenyan purple tea products (aerated/black and unaerated/green) have delphinidin, cyanidin, pelargonidin, peonidin and malvidin as the dominant anthocyanidins [33]. These studies illustrate that the composition of anthocyanins in purple tea is primarily determined by genotype in addition to the effect of environment and processing conditions. In the current study, in addition to the major 10 anthocyanin compounds, we were able to detect other minor anthocyanin pigments in purple tea (Supplementary Table S1). In addition to the three main anthocyanindins (delphinidin, cyanidin and petunidin), pelargonidin was also detected. These minor anthocyanin compounds were found at very low concentrations and could vary from one year to another, subject to environmental factors. No anthocyanins were detected in green tea (Supplementary Figure S2).

Anthocyanins were present in purple tea leaves at much higher concentrations (Table 2) compared with their corresponding flakes (Table 3). The water extraction and flaking process resulted in substantial reductions in anthocyanins in purple tea flakes, most likely due to thermal degradation and oxidation of anthocyanins. Delphinidin-coumaroyl-hexoside was the major anthocyanin pigment in purple tea (2497–3228 µg/g) followed by delphinidin-3-galactoside (584–918 µg/g), while cyanidin-coumaroyl-hexoside came third (506–653 µg/g) and delphinidin-3-glucoside (294–478 µg/g) was fourth. The gross total of anthocyanins significantly varied among the lots, ranging from 5045–7036 µg/g in purple tea leaves compared with 70.9–457.8 µg/g in purple tea flakes. This indicates that some of the anthocyanin compounds are lost and/or oxidized during processing. Despite the significant reduction in anthocyanins, the flakes can still be considered a good source of anthocyanin pigments. Since anthocyanins are considered health-enhancing substances, purple tea flakes would have potential beneficial health effects such as improved bioavailability due to their extractability and solubility in water. The results have shown that delphinidin-based anthocyanins are the dominant pigments in purple tea, constituting more than 78% of the total major anthocyanins. Delphinidin has a broad therapeutic spectrum including anticancer activity against a variety of cancers and synergistic effects against cancer when used in combination with some clinically used drugs and potent cardio-protective agents, with effects against psoriasis, osteoporosis and a variety of viral species [36]. Several mechanisms are involved in the preventive and protective roles of delphinidin, such as scavenging of free radicals, interfering with protein targets of the PI3K/Akt/mTOR, MAPK, and ubiquitin-proteasome pathways, lowering the expression of NOX, cytokines, mucins, MMPs, and STATs, inhibiting of ACE, hindering the entry of viral particles and enhancing the secretion of insulin, GLP-1, LCE3 genes and certain epidermal proteins to exert beneficial effects on the gut microbiome population. Delphinidin in the form of anthocyanin or anthocyanidin has been used along with other nutraceuticals or as a dietary supplement to promote health. In this regard, purple tea flakes could be employed as a good source of delphinidin in addition to their content of catechins, protein and minerals.

### 3.3. Composition of Catechins

**Figure 4:**
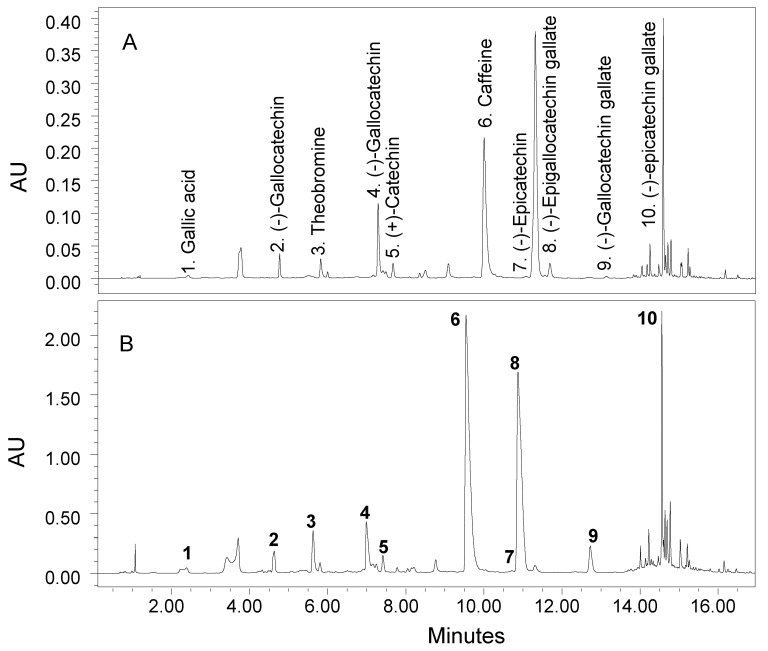
Typical UPLC chromatograms of catechin compounds in green tea leaves (**A**) and flakes (**B**).

Catechins are polyphenolic compounds found abundantly in green tea, red wine, chocolate and fresh fruits such as apricots and cherries. They are dietary antioxidants which can elicit several positive functions in the area of obesity, diabetes, some cancers and others. Catechin-rich ingredients have the potential for use in functional food, cosmetics and healthcare industries either for increasing the shelf life of products or health benefits for consumers. The composition of catechins in purple tea leaves and flakes are presented in Figure 2B,C in comparison with green tea leaves and flakes (Figure 4A,B), and their concentrations are given in Table 4 and Table 5, respectively. Both teas are good sources of catechins at levels of 140–148 and 219–263 mg/g in purple tea leaves and flakes, respectively, compared with 259–311 and 416–426 mg/g in green tea products. In general, green tea contains higher concentrations of catechins than purple tea. The main catechins in purple tea were epigallocatechin gallate (EGCG), epicatechin gallate (ECG) and epigallocatechin (EGC), with EGCG being the most abundant at concentrations of 119 and 125 mg/g in leaves and flakes, respectively. Small concentrations of gallocatecin (GC), catechin (C), and epicatechin (EC) were also found in purple tea. Similarly, EGCG was the main catechin in green tea leaves at a concentration of 119–151 mg/g. EGC came second followed by GC and EPG. Other studies have shown that EGCG is the major catechin in green tea [15,37]. The content of catechins in purple and green tea flakes was higher than that in their corresponding tea leaves since processing of tea leaves into flakes resulted in concentrating catechins in the flakes. Additionally, the composition of catechins underwent changes during processing, with EGCG, GCG, ECG and EC becoming the dominant components in the flakes. There were considerable increases in catechins in processed flakes, especially GCG. Several factors can affect the stability of catechins during processing, such as pH, temperature, oxygen, antioxidants, metal ions and the concentration of other tea polyphenols [38,39,40]. It has been reported that brewing at 85 °C for 3–5 min increases epicatechins but increasing the brewing time longer results in a decrease in their content. Furthermore, the amount of non-episcatechins continued to increase with a longer extraction time [40]. The amount of GCG exceptionally increased in the purple and green tea flakes, e.g., from non-detected to 10.2–69.9 mg/g and from 0.9–1.3 to 23.5–24.4 mg/g, respectively, possibly due to epimerization of EGCG. When green tea drink or pure EGCG were autoclaved at 120 °C for 20 min, the epimerization of EGCG to GCG was detected [38]. A fair portion of EGCG (and related catechins) in green tea leaves is epimerized into GCG during the brewing process, which has been found to be able to inhibit human topoisomerase IIα and Iiβ, as EGCG does [41]. It seems that both configurations (*trans* and *cis*) have similar biological effects. In addition, EGCG could also be oxidized into EGCG dimers, and the reaction rate is dependent on several factors such as temperature, pH, oxygen, concentration of EGCG and the presence of antioxidants [40]. Analysis of tea samples taken from the same plant but manufactured by different degrees of fermentation has shown that the level of EGCG and catechins is in the order green tea > oolong tea > black tea [42]. Thus, each tea drink has a unique composition of catechins which impacts its health effects. There were significant differences in the concentration of catechins among the tea lots, probably due to differences in their content of catechins in the original materials. The high content of catechins and anthocyanins could make purple tea leaves a healthy drink and the flakes a promising ingredient for nutraceutical, cosmetic and healthcare product applications.

### 3.4. Other Bioactive Compounds

Three other bioactive components including gallic acid, theobromine and caffeine were also detected in purple and green tea products as part of the catechins analysis (Figure 2B,C and Figure 4A,B). Gallic acid (3,4,5-trihydroxybenzoic acid) is a naturally occurring polyphenolic compound widely present in fruits and nuts that has demonstrated several positive functions such as antioxidant, anti-inflammatory and anticancer [43,44]. Purple tea leaves and flakes had higher contents of gallic acid compared with green tea products, e.g., 5.8 and 39.9 mg/g versus 1.8 and 9.9 mg/g, respectively (Table 6). The flakes contained much higher concentrations of gallic acid than leaves in both tea types. The increase in gallic acid during processing of leaves into flakes could be attributed to the degradation of catechins and other polyphenols during thermal processing.

Theobromine is a bitter alkaloid primary found in cocoa and chocolate. It can improve circulatory, respiratory and excretory systems in humans, but long-term intake of theobromine (e.g., 1500 mg/day) may cause nausea, headache, bad mood and loss of appetite. In other words, it can contribute positively or negatively to the effect of chocolate on mood subject to its dose [45]. At high doses (>250 mg), it has negative effects on mood and increases heart rate. Again, purple tea leaves and flakes had higher amounts of theobromine than green tea products, e.g., 7.4 and 16.7 mg/g versus 1.3 and 4.1 mg/g, respectively (Table 6). Once again, the flakes from both teas exhibited higher concentrations of theobromine in comparison with tea leaves. The extraction and thermal processes considerably increased theobromine in the flakes, possibly due to the degradation of other bioactive compounds.

Caffeine is a bitter alkaloid primary found in coffee, tea and cola. Both theobromine and caffeine are related since both are methylxanthine derivatives. Caffeine stimulates the central nervous system; thus, it is most commonly used to improve mental alertness. Unlike gallic acid and theobromine, caffeine content was higher in green tea leaves and flakes compared with purple tea leaves and flakes, e.g., 18.4 and 54.5 mg/g versus 14.9 and 37.3 mg/g, respectively (Table 6). The flakes contained higher amounts of caffeine compared with leaves for both purple and green teas. Water extraction and flaking of tea leaves resulted in an increase in caffeine and theobromine probably due to enzymatic conversion and/or thermal degradation. Caffeine was assessed in 31 commercial teas ranging from 12.4 to 33.4 mg/g, and the level of caffeine is in the order of green tea > oolong tea > black tea [42], which is within the range of caffeine in green tea in the current study. In general, purple tea can be considered as a promising source of anthocyanins, catechins, gallic acid, theobromine and caffeine. This indicates the potential of purple tea flakes in the cosmetics and healthcare product industries.

## 4. Conclusions

Purple tea holds promise in making healthy tea drinks and functional ingredients for the beverage, cosmetics and healthcare industries due to its reasonable contents of anthocyanins in addition to catechins and other bioactive components. Green tea contained catechins at higher levels than purple tea, but no anthocyanins were found in green tea products. Other bioactive compounds including gallic acid, theobromine and caffeine were also present in purple and green tea. The presence of a diverse array of bioactive compounds, primarily polyphenols, in purple tea in addition to a high content of protein and minerals could make it a good choice for several food and non-food applications. In particular, purple tea flakes hold great potential for the cosmetics and healthcare industries due to their solubility in water. It would be of interest to study antioxidant properties and bioavailability of anthocyanins and catechins in purple tea brews and flakes to determine their contribution to daily intake and potential health benefits.

## Figures and Tables

**Table 1:** Moisture, total ash and protein content of purple and green tea leaves and flakes ^x^.

Tea Product	Moisture (%)	Ash (% As Is)	Protein (% As Is)
Purple tea leaves-lot 1	6.21 ± 0.06 ^a^	5.39 ± 0.05 ^a^	24.97 ± 0.06 ^a^
Purple tea leaves-lot 2	6.34 ± 0.02 ^a^	5.18 ± 0.06 ^a^	25.70 ± 0.05 ^a^
Purple tea leaves-lot 3	6.46 ± 0.01 ^a^	5.16 ± 0.02 ^a^	25.47 ± 0.04 ^a^
Mean	6.34	5.24	25.38
Purple tea flakes-lot 1	6.59 ± 0.05 ^a^	7.94 ± 0.04 ^b^	20.12 ± 0.06 ^ab^
Purple tea flakes-lot 2	5.74 ± 0.01 ^a^	8.38 ± 0.02 ^ab^	19.48 ± 0.15 ^b^
Purple tea flakes-lot 3	5.71 ± 0.01 ^a^	9.49 ± 0.04 ^a^	20.93 ± 0.07 ^a^
Mean	6.01	8.60	20.18
Green tea leaves-lot 1	6.08 ± 0.04 ^a^	5.31 ± 0.03 ^a^	22.19 ± 0.17 ^a^
Green tea leaves-lot 2	6.07 ± 0.02 ^a^	5.33 ± 0.01 ^a^	18.88 ± 0.11 ^b^
Mean	6.08	5.32	20.54
Green tea flakes-lot 1	6.01 ± 0.01 ^a^	9.22 ± 0.02 ^a^	17.62 ± 0.07 ^a^
Green tea flakes-lot 2	5.75 ± 0.03 ^a^	9.21 ± 0.01 ^a^	17.31 ± 0.29 ^a^
Mean	5.88	9.22	17.47

^x^ For each tea product, mean values followed by a different superscript letter are significantly different between lots at *p* < 0.05.

**Table 2:** Chromatographic and mass characteristics and concentration of major anthocyanin pigments in purple tea leaves.

Peak Number ^w^	RT (min)	Major Ions (*m/z*)	λ_max _ (nm)	Identity of Anthocyanin ^y^	Concentration (Mean ± SD, µg/g) ^z^		
					Lot 1	Lot 2	Lot 3
1	5.8	465/303	525	Dp-3-Gal	918 ± 10.6 ^a^	584 ± 3.8 ^b^	628 ± 5.3 ^b^
2	6.4	465/303	524	Dp-3-Glu	478 ± 10.1 ^a^	294 ± 6.4 ^b^	319 ± 2.1 ^b^
3	7.0	449/287	517	Cy-3-Gal	348 ± 0.4 ^a^	219 ± 0.9 ^b^	230 ± 1.8 ^b^
4	7.6	449/287	516	Cy-3-Glu	161 ± 7.0 ^a^	97 ± 4.5 ^b^	103 ± 0.6 ^b^
5	12.7	611/303	530	Dp-Cou-Hex	378 ± 18.2 ^a^	202 ± 3.1 ^b^	237 ± 1.3 ^b^
6	14.0	611/303	532	Dp-Cou-Hex	3228 ± 5.2 ^a^	2497 ± 31.2 ^b^	2634 ± 15.6 ^b^
7	15.1	611/303	528	Dp-Cou-Hex	141 ± 3.1 ^a^	109 ± 3.8 ^b^	117 ± 0.3 ^b^
8	15.4	595/287	520	Cy-Cou-Hex	653 ± 1.4 ^a^	506 ± 1.8 ^b^	569 ± 0.1 ^b^
9	15.9	625/317	533	Pt-Cou-Hex	160 ± 1.0 ^a^	93 ± 2.8 ^b^	97 ± 5.9 ^b^
10	16.7	595/287	520	Cy-Cou-Hex	122 ± 4.2 ^a^	100 ± 0.2 ^b^	106 ± 2.2 ^b^
Total major	-	-	-	-	6587	4701	5040
Total minor	-	-	-	-	449	344	319
Gross total ^x^	-	-	-	-	7036	5045	5359

^w^ Peak numbers match those in Figure 1. ^x^ Gross total includes major and minor anthocyanin compounds detected in tea leaves. ^y^ Cou, coumaroyl; Cy, cyanidin; Dp, delphinidin; Gal, galactoside; Glu, glucoside; Hex, Hexoside; Pt, petunidin. ^z^ Mean values followed by a different superscript letter are significantly different between lots at *p* < 0.05.

**Table 3:** Concentration of major anthocyanin pigments in purple tea flakes.

Peak Number ^w^	Identity of Anthocyanin ^y^	Concentration (Mean ± SD, µg/g) ^z^		
		Lot 1	Lot 2	Lot 3
1	Dp-3-Gal	49.9 ± 0.1 ^a^	18.6 ± 0.7 ^b^	11.0 ± 0.7 ^c^
2	Dp-3-Glu	37.1 ± 0.3 ^a^	13.1 ± 0.6 ^b^	9.7 ± 0.4 ^b^
3	Cy-3-Gal	15.1 ± 0.3 ^a^	6.2 ± 0.3 ^b^	2.6 ± 0.1 ^c^
4	Cy-3-Glu	10.8 ± 0.3 ^a^	4.1 ± 0.2 ^b^	2.8 ± 0.1 ^b^
5	Dp-Cou-Hex	20.8 ± 0.6 ^a^	9.0 ± 0.4 ^b^	5.0 ± 0.2 ^c^
6	Dp-Cou-Hex	234.3 ± 2.5 ^a^	15.9 ± 1.1 ^b^	24.5 ± 0.3 ^c^
7	Dp-Cou-Hex	13.5 ± 0.2 ^a^	2.7 ± 0.1 ^b^	3.6 ± 0.2 ^b^
8	Cy-Cou-Hex	47.5 ± 0.3 ^a^	3.7 ± 0.1 ^b^	5.2 ± 0.3 ^b^
9	Pt-Cou-Hex	12.7 ± 0.2 ^a^	2.5 ± 0.1 ^b^	3.2 ± 0.2 ^b^
10	Cy-Cou-Hex	10.6 ± 0.4 ^a^	2.8 ± 0.1 ^b^	3.1 ± 0.2 ^b^
Total major	-	425	78.4	70.2
Total minor	-	5.8	0.7	0.7
Gross total ^x^	-	457.8	79	70.9

^w^ Peak numbers match those in Figure 1. ^x^ Gross total includes major and minor anthocyanin compounds detected in tea flakes. ^y^ Cou, coumaroyl; Cy, cyanidin; Dp, delphinidin; Gal, galactoside; Glu, glucoside; Hex, hexoside; Pt, petunidin. ^z^ Mean values followed by a different superscript letter are significantly different between lots at *p* < 0.05.

**Table 4:** Composition of catechin compounds in purple tea leaves and flakes.

Catechin	Peak Number ^x^	Concentration (Mean ± SD, mg/g) ^y^		
		Lot 1	Lot 2	Lot 3
Purple tea leaves				
(−)-Gallocatechin	2	1.73 ± 0.02 ^a^	2.07 ± 0.01 ^a^	1.83 ± 0.02 ^a^
(−)-Epigallocatechin	4	16.36 ± 0.19 ^a^	14.37 ± 0.21 ^b^	12.30 ± 0.15 ^c^
(+)-Catechin	5	0.23 ± 0.01 ^b^	0.40 ± 0.01 ^a^	0.47 ± 0.03 ^a^
(−)-Epicatechin	7	5.17 ± 0.17 ^a^	5.16 ± 0.29 ^a^	5.33 ± 0.11 ^a^
(−)-Epigallocatechin gallate	8	89.46 ± 0.43 ^b^	95.07 ± 0.25 ^a^	96.51 ± 0.24 ^a^
(−)-Gallocatechin gallate	9	nd	nd	nd
(−)-Epicatechin gallate	10	27.45 ± 0.10 ^b^	28.09 ± 1.13 ^b^	31.03 ± 1.81 ^a^
Total	-	140.4	145.2	147.5
Purple tea flakes				
(−)-Gallocatechin	2	8.74 ± 0.42 ^b^	15.70 ± 0.26 ^a^	2.73 ± 0.11 ^c^
(−)-Epigallocatechin	4	10.61 ± 0.01 ^c^	48.54 ± 0.62 ^a^	23.72 ± 0.03 ^b^
(+)-Catechin	5	4.49 ± 0.02 ^a^	4.43 ± 0.04 ^a^	2.09 ± 0.01 ^a^
(−)-Epicatechin	7	8.72 ± 0.38 ^c^	73.95 ± 0.55 ^a^	66.95 ± 0.61 ^b^
(−)-Epigallocatechin gallate	8	85.40 ± 0.76 ^b^	113.80 ± 3.17 ^a^	111.05 ± 3.86 ^a^
(−)-Gallocatechin gallate	9	69.85 ± 1.15 ^a^	38.90±0.22 ^b^	10.15 ± 0.18 ^c^
(−)-Epicatechin gallate	10	31.11 ± 0.77 ^b^	45.78 ± 0.41 ^a^	46.02 ± 0.12 ^a^
Total	-	218.9	341.05	262.7

^x^ Peak numbers match those in Figure 3. ^y^ Mean values followed by a different superscript letter are significantly different between lots at *p* < 0.05.

**Table 5:** Composition of catechin compounds in green tea leaves and flakes.

Catechin	Peak Number ^x^	Concentration (Mean ± SD, mg/g) ^y^	
		Lot 1	Lot 2
Green tea leaves			
(−)-Gallocatechin	2	27.89 ± 0.28 ^a^	31.35 ± 0.47 ^b^
(−)-Epigallocatechin	4	74.88 ± 0.83 ^b^	83.41 ± 1.26 ^a^
(+)-Catechin	5	3.47 ± 0.06 ^a^	3.73 ± 0.04 ^a^
(−)-Epicatechin	7	13.14 ± 0.06 ^a^	13.63 ± 0.50 ^a^
(−)-Epigallocatechin gallate	8	118.5 ± 0.93 ^b^	150.5 ± 0.86 ^a^
(−)-Gallocatechin gallate	9	0.90 ± 0.10 ^b^	1.31 ± 0.04 ^a^
(−)-Epicatechin gallate	10	20.41 ± 1.11 ^b^	27.05 ± 0.21 ^a^
Total	-	259.2	311.0
Green tea powders			
(−)-Gallocatechin	2	61.70 ± 0.42 ^a^	60.45 ± 0.35 ^a^
(−)-Epigallocatechin	4	92.65 ± 0.66 ^a^	88.80 ± 0.19 ^a^
(+)-Catechin	5	5.78 ± 0.12 ^a^	5.94 ± 0.03 ^a^
(−)-Epicatechin	7	76.30 ± 0.81 ^a^	71.60 ± 0.52 ^a^
(−)-Epigallocatechin gallate	8	124.80 ± 4.46 ^b^	142.60 ± 4.71 ^a^
(−)-Gallocatechin gallate	9	23.54 ± 0.07 ^a^	24.37 ± 0.11 ^a^
(−)-Epicatechin gallate	10	31.42 ± 0.42 ^a^	31.71 ± 0.76 ^a^
Total	-	416.2	425.5

^x^ Peak numbers match those in Figure 3. ^y^ Mean values followed by a different superscript letter are significantly different between lots at *p* < 0.05.

**Table 6:** Concentration of other bioactive compounds found in purple and green tea leaves and flakes (mean ± SD, mg/g) ^x^.

Tea	Gallic Acid (Peak #1) ^y^	Theobromine (Peak #3) ^y^	Caffeine (Peak #6) ^y^
Purple tea leaves-lot 1	4.77 ± 0.13 ^b^	5.01 ± 0.06 ^b^	15.35 ± 0.07 ^a^
Purple tea leaves-lot 2	6.54 ± 0.04 ^a^	9.12 ± 0.07 ^a^	15.53 ± 0.32 ^a^
Purple tea leaves-lot 3	6.12 ± 0.11 ^a^	8.03 ± 0.03 ^a^	13.83 ± 0.21 ^b^
Average	5.81	7.39	14.90
Purple tea flakes-lot 1	38.95 ± 1.17 ^b^	14.71 ± 0.33 ^b^	30.18 ± 0.07 ^c^
Purple tea flakes-lot 2	37.35 ± 0.19 ^b^	13.87 ± 0.47 ^b^	43.30 ± 0.18 ^a^
Purple tea flakes-lot 3	43.37 ± 0.09 ^a^	21.38 ± 0.34 ^a^	38.51 ± 0.17 ^b^
Average	39.89	16.65	37.33
Green tea leaves-lot 1	1.69 ± 0.05 ^a^	1.24 ± 0.01 ^a^	17.12 ± 0.16 ^b^
Green tea leaves-lot 2	1.98 ± 0.01 ^a^	1.35 ± 0.06 ^a^	19.74 ± 0.13 ^a^
Average	1.84	1.30	18.43
Green tea flakes-lot 1	10.08 ± 0.03 ^a^	4.27 ± 0.18 ^a^	56.00 ± 0.52 ^a^
Green tea flakes-lot 2	9.66 ± 0.05 ^a^	4.14 ± 0.06 ^a^	52.90 ± 0.55 ^b^
Average	9.87	4.07	54.45

^x^ For each tea product, mean values in a column followed by a different superscript letter are significantly different between lots at *p* < 0.05. ^y^ Peak numbers match those in Figure 3.

## Data Availability

Data is contained within the article and supplementary materials.

## Supplementary Materials

The following supporting information can be downloaded at: https://www.mdpi.com/article/10.3390/molecules27196676/s1, Supplementary Table S1. Minor anthocyanin pigments detected in purple tea; Supplementary Figure S1. MS fragmentation spectra of (A) peak 1 (Dp-3-Gal), (B), peak 3 (Cy-3-Gal), (C) peak 7 (Dp-Cou-Hex) and (D) peak 9 (Pt-Cou-Hex); Supplementary Figure S2. Typical UPLC chromatograms of green tea leaves (A) and flakes (B) showing no anthocyanin peaks.

## Sample Availability

Samples and chemical compounds are available from the supplies listed in the article.
