# Supplementary material for: Identification and Quantification of Anthocyanin and Catechin Compounds in Purple Tea Leaves and Flakes
Source: Molecules. 2022 Oct 7;27(19):6676. doi: 10.3390/molecules27196676 (PMC9570566; doi:10.3390/molecules27196676)
Supplement: Supplementary file 1 [file molecules-27-06676-s001.zip › molecules-1915117-supplementary.pdf]

Supplementary Table S1

Minor anthocyanin pigments detected in purple tea

RT (min)	Identity of anthocyanin	Major ions (*m/z*)	
		Anthocyanin	Anthocyanidin base
8.1	Pt-Hex	479.1167	317.0648
8.5	Pl-Hex	433.1124	271.0598
8.7	Pt-Hex	479.1167	317.0648
14.6	Cy-Cou-Hex	595.1439	287.0544
15.1	Pt-Cou-Hex	625.1547	317.0648
15.9	Pl-Cou-Hex	579.1491	271.0592
17.5	Pl-Cou-Hex	579.1491	271.0592
17.9	Pt-Cou-Hex	625.1547	317.0649
19.3	Pl-Cou-Hex	579.1491	271.0592

[y]Cou, coumaroyl; Cy, cyanidin; Hex, Hexoside; Pl-Pelargonidin; Pt, petunidin.

Supplementary Figure S1

MS fragmentation spectra of (A) peak 1 (Dp-3-Gal), (B), peak 3 (Cy-3-Gal), (C) peak 7 (Dp-Cou-Hex) and (D) peak 9 (Pt-Cou-Hex).

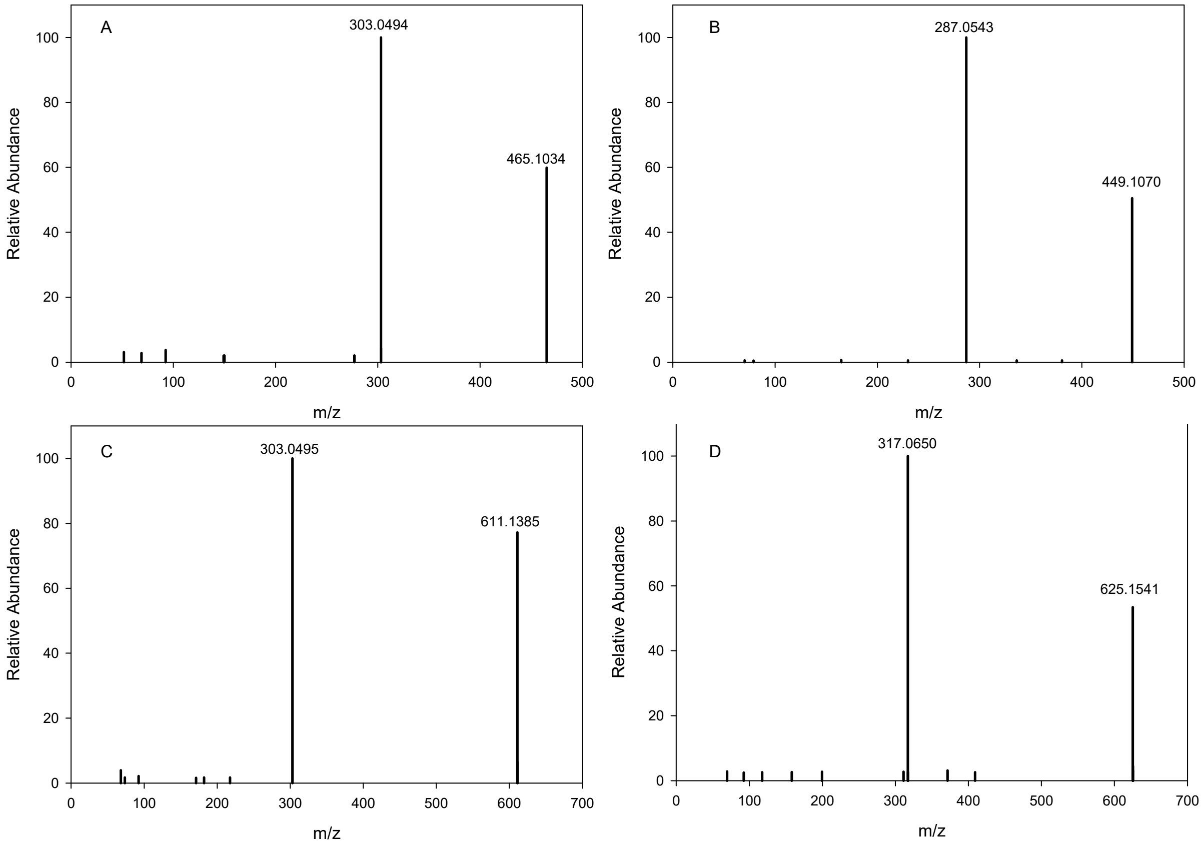

Supplementary Figure S2

Typical UPLC chromatograms of green tea leaves (A) and flakes (B) showing no anthocyanin peaks.

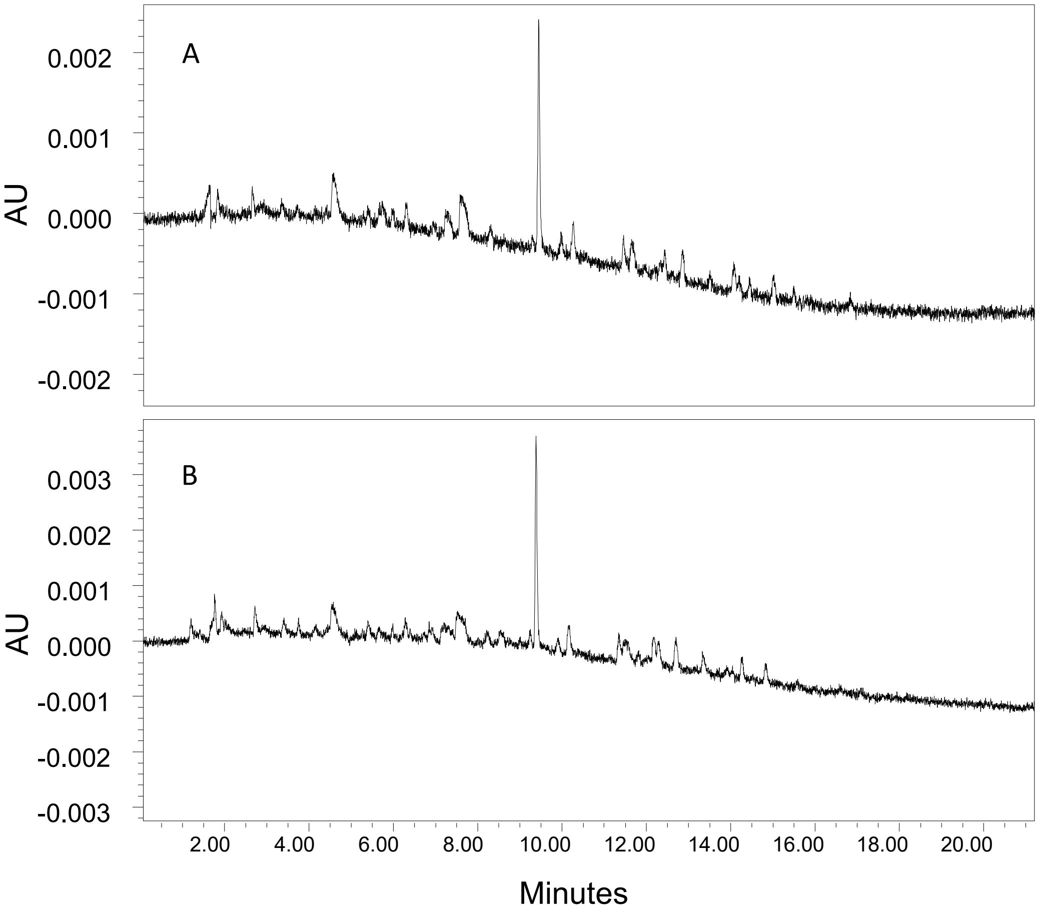

Supplementary Table S1. Minor anthocyanin pigments detected in purple tea
Supplementary Figure S1. MS fragmentation spectra of (A) peak 1 (Dp-3-Gal), (B), peak 3 (Cy-3-Gal), (C) peak 7 (Dp-Cou-Hex) and (D) peak 9 (Pt-Cou-Hex).
Supplementary Figure S2. Typical UPLC chromatograms of green tea leaves (A) and flakes (B) showing no anthocyanin peaks.
